# Presence of Arteriovenous Communication between Left Testicular Vessels and Its Clinical Significance

**DOI:** 10.1155/2014/160824

**Published:** 2014-03-04

**Authors:** Naveen Kumar, Ravindra Swamy, Jyothsna Patil, Anitha Guru, Ashwini Aithal, Prakashchandra Shetty

**Affiliations:** ^1^Melaka Manipal Medical College (Manipal Campus), Manipal University, Manipal 576 104, India; ^2^Department of Anatomy, Melaka Manipal Medical College (Manipal Campus), Manipal University, Manipal 576 104, India

## Abstract

Maintenance of testicular temperature below body temperature is essential for the process of spermatogenesis. This process of thermoregulation is mainly achieved by testicular veins through pampiniform venous plexus of the testis by absorbing the heat conveyed by the testicular arteries. However, this mechanism of thermoregulation may be hampered if an abnormal communication exists between the testicular vessels. We report herewith a rare case of arteriovenous communication between testicular artery and testicular vein on left side. The calibre of the communicating vessel was almost similar to left testicular artery. Such abnormal communication may obstruct the flow of blood in the vein by causing impairment in the perfusion pressure with the eventual high risk of varicocele.

## 1. Introduction

Testicular arteries are long, slender vessels originating from abdominal aorta slightly below the origin of renal arteries. Testicular arteries reach the deep inguinal ring and pass through spermatic cord in the inguinal canal and finally enter the scrotum to supply testis [1]. Venules draining the testes join in the mediastinum testes to form several veins. In the spermatic cord these veins form the pampiniform plexus—an intercommunicating venous network that surrounds the testicular artery and cools arterial blood before it reaches the testes. The right testicular vein drains into the inferior vena cava and the left testicular vein drains into the left renal vein.

Maintaining the suitable temperature for the crucial stages of spermatogenesis is priority of the body. The specialised venous network in the form of pampiniform plexus of the testicular veins allows countercurrent heat exchange with the testicular artery and maintains the thermoregulation.

Occasionally there may be parallel collaterals to the gonadal veins at different locations of its extent [2]. Very rarely additional collateral supply can occur from retroperitoneal branches which in turn communicate with contralateral gonadal vein or renal veins [3]. But, the arteriovenous communication between the testicular artery and vein is the rarest of all variations pertaining to the vascular anomalies affecting the gonadal vasculature. We report herewith one such exceptional case with the discussion of possible complications having arteriovenous communication between left testicular vessels.

## 2. Case Report

**Figure 1:**
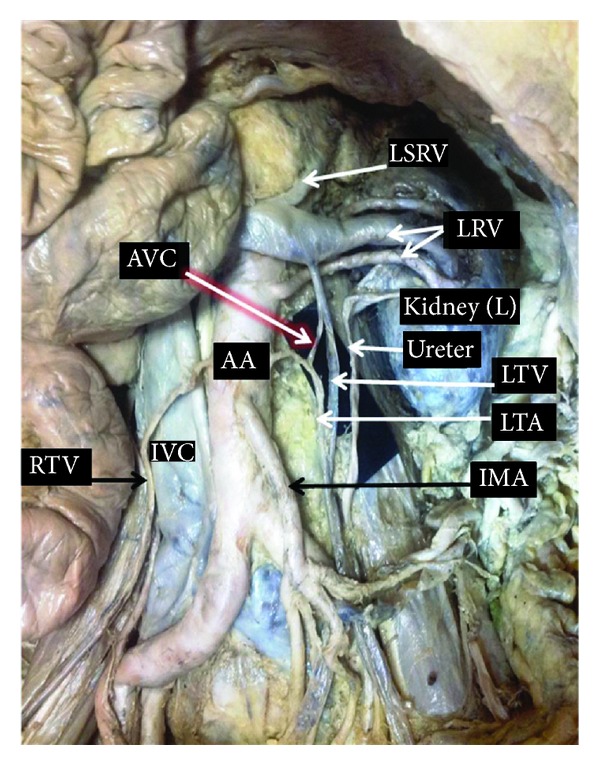
Showing presence of arteriovenous communication (AVC) between left testicular artery (LTA) and Left testicular vein (LTV). Right testicular vessels (RTV) were found to be normal. LRV: left renal vessels, LSRV: left suprarenal vein, IMA: inferior mesenteric artery, RTV: right testicular vessels, AA: abdominal aorta, and IVC: inferior vena cava.

During routine cadaveric dissection for the undergraduate medical students, we observed a prominent communicating vascular channel between testicular artery and testicular vein on the left side of the male adult cadaver aged about 65 years. The calibre of the communicating vessel was almost similar to testicular artery. The communicating channel was found to be connecting the vessels between 1.5 and 2 inches at the commencement of the testicular artery and termination of testicular vein (Figure 1). The skin from the superficial inguinal opening up to the scrotum was removed and the spermatic cord was observed. The scrotum and testis were also exposed and observed. No apparent anomaly was found in the pampiniform plexus. On the right side there was no such communication between right testicular vein and artery.

## 3. Discussion

Variation in testicular artery and vein is not uncommon. Studies show that the testicular artery variations are more common on right side as compared to left side [4]. Cicekcibai et al. observed that origin of the gonadal artery from the renal artery was found in 5.5% of cases [5]. Origin of testicular artery from a high level was found in a study done by Rusu and Mamatha et al. [6, 7]. Variation of testicular vein was found in 21.3% of cadavers and to be more common on the left side according to a study conducted by Asala et al. [8]. A study revealed that variation in the number of the right testicular vein was found in 15% of specimens and variations in number of left testicular vein were in 18% of specimens [9]. The termination of the right testicular vein into right renal vein, accessory renal vein, or lower part of the inferior vena cava was also found [4, 10–12].

The present case is second extremely rare report of arteriovenous communication between the left testicular artery and the vein. Only one case report by Nayak et al. has been found in literature regarding such variation [13]. There is striking similarity between the present case and case reported by Nayak et al. in which the left testicular artery originated from the lower part of the abdominal aorta and a communicating vessel joining the left testicular artery with the left testicular vein was observed [13]. The only difference is that in the present case the right testicular vein is normal, while Nayak et al. mentioned a bifurcated right testicular vein at its termination. Further we found that the left testis and the left pampiniform plexus were apparently normal in the present case. Communicating vessel between the testicular artery and vein of the same side may lead to blood flow in either direction. Blood may flow from the artery into the vein, thus increasing the venous pressure in testicular vein which may become one of the causes for varicocele. If blood from vein enters into artery then it might affect the nutritional supply to the testis and affect the functional capability of testis. This arteriovenous communication may affect the thermoregulation of the testis,thus affecting the process of spermatogenesis [13]. Spermatogenesis requires maintenance of testicular temperature below the body temperature. Thermoregulation of testis is achieved by testicular veins as they form pampiniform venous plexus around the testicular artery and thus absorb the heat conveyed by the testicular arteries. This mechanism of thermoregulation may be hampered when there is abnormal communication between the testicular vessels, thus affecting the spermatogenesis.

Etiological factors of varicocele and male infertility are numerous and in rare cases it is difficult for the clinician to pinpoint the exact cause of the disease. Testicular arteriovenous communication can be one of the etiological causes for the abovementioned diseases. Even though the cases of testicular arteriovenous communication are rare, cases of male infertility due to oligospermia and varicocele should be investigated with the help of Doppler ultrasound and arteriogram to rule out any vascular anomaly such as described in the present case. During the procedure for varicocele embolisation to treat varicocele on left side, the catheter is passed through the renal vein and then into testicular vein. The catheter or probe during such procedure may accidently pass in the testicular arteriovenous communication reaching the testicular artery. Thus during left varicocele embolisation, presence of testicular arteriovenous communication should be noted.

## 4. Conclusion 

Arteriovenous communication of testicular vessels should be kept in mind by the clinicians and surgeons treating the varicocele and male infertility.
